# Supplementary figures and images for: A new Amazonian species of Allobates Zimmermann & Zimmermann, 1988 (Aromobatidae) with a trilled advertisement call
Source: PeerJ. 2022 Mar 7;10:e13026. doi: 10.7717/peerj.13026 (PMC8908894; doi:10.7717/peerj.13026)

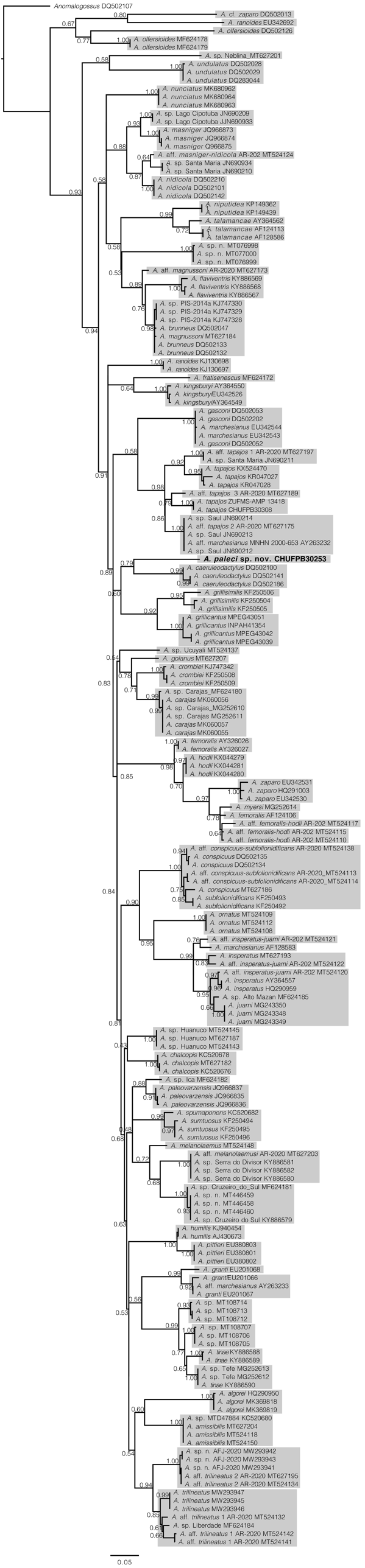

Supplement: Supplemental Information 2 — Nodes are labeled with the bootstrap support. Gray areas represent each evolutionary entity delimited by PTP. [file peerj-10-13026-s002.jpg]

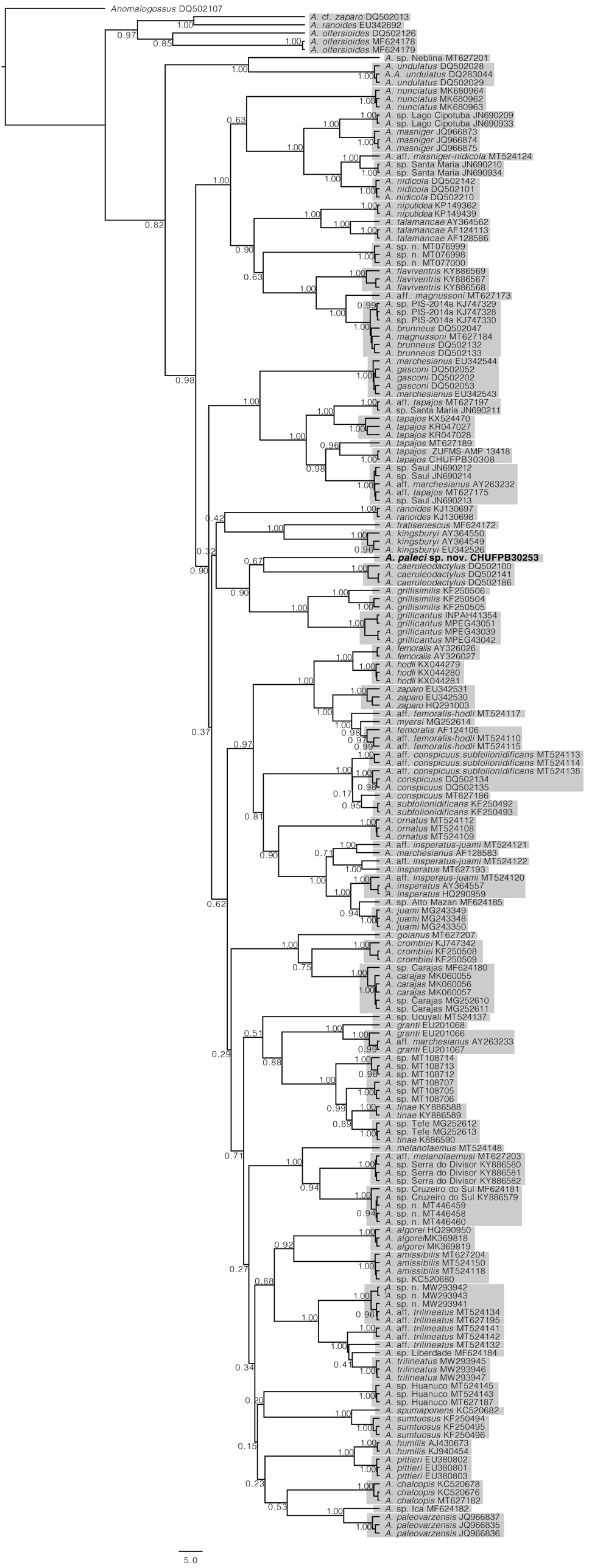

Supplement: Supplemental Information 3 — Nodes are labeled with the Bayesian posterior probability. Scale bar in million years. Gray areas represent each evolutionary entity delimited by GMYC. [file peerj-10-13026-s003.jpg]
