# Supplementary material for: A new Amazonian species of Allobates Zimmermann & Zimmermann, 1988 (Aromobatidae) with a trilled advertisement call
Source: PeerJ. 2022 Mar 7;10:e13026. doi: 10.7717/peerj.13026 (PMC8908894; doi:10.7717/peerj.13026)
Supplement: Supplemental Information 8 [file peerj-10-13026-s008.docx]

**Appendix 1**

*Allobates paleci* sp. nov. = Jacareacanga-PA: CHUFPB30242, CHUFPB30244–45, CHUFPB30248, CHUFPB30251–53, CHUFPB30256, CHUFPB30281.

*Allobates* aff. *tapajos* = Jacareacanga-PA: CHUFPB30308; ZUFMS7723–29; Paranaíta-MT: ZUFMS7720–22, ZUFMS10774–77.
